# Underdiagnosis of iron deficiency anemia among patients with colorectal cancer: an examination of electronic medical records

**DOI:** 10.1186/s12885-022-09542-z

**Published:** 2022-04-21

**Authors:** Trishnee Bhurosy, Anika Jishan, Patrick M. Boland, Yen-Han Lee, Carolyn J. Heckman

**Affiliations:** 1 Department of Population Health, Hofstra University, Hempstead, New York 11549 USA; 2 Section of Behavioral Sciences, Rutgers Cancer Institute of New Jersey, 195 Little Albany Street, New Brunswick, NJ 08901 USA; 3 Division of Medical Oncology, Rutgers Cancer Institute of New Jersey, 195 Little Albany Street, New Brunswick, NJ 08901 USA; 4 Department of Public Health and Sports Medicine, Missouri State University, Springfield, MO 65897 USA

**Keywords:** Screening, Iron deficiency anemia, Colorectal cancer, Electronic medical record

## Abstract

**Background:**

Timely diagnosis and management of iron deficiency anemia (IDA) in colorectal cancer (CRC) patients improves overall quality of life and survival. This study assessed the proportion of CRC patients who were formally diagnosed with IDA and factors that predict a formal diagnosis of IDA and receiving iron therapy.

**Methods:**

We retrieved electronic medical records (EMRs) of CRC patients from a large comprehensive cancer center in the Northeastern part of the United States (*n* = 499). We abstracted sociodemographic characteristics, relevant laboratory results, IDA diagnosis, and iron supplementation from the EMRs. We assessed relationships between participant characteristics, a diagnosis of IDA and receiving iron therapy through adjusted logistic regressions.

**Results:**

IDA was formally diagnosed in 26 (5.2%) individuals judged by EMR documentation. Only 153 (30.7%) participants had iron laboratory results available. Among the 153 patients with iron panel data available, 113 (73.9%) had iron deficiency. Seventy-six had absolute iron deficiency as shown by ferritin levels below 100 ng/mL and iron saturation less than 20% and 37 had functional iron deficiency as shown by ferritin levels between 100 and 500 ng/mL and iron saturation less than 20%. 12% of all patients had documentation of iron therapy receipt. A formal diagnosis of IDA was not associated with any of the covariates.

**Conclusions:**

Iron deficiency anemia is under-diagnosed among CRC patients and most likely under-documented in clinical notes. Rates of iron repletion are low, suggesting that many patients with IDA are untreated. Future research should explore provider-level and other strategies for improving assessment and diagnosis of IDA among CRC patients.

## Background

Iron deficiency anemia (IDA) remains the top cause of anemia worldwide [1]. Nearly two-thirds of cancer patients are likely to develop anemia, with IDA as the cause or a contributing component in 60% of cases [2]. In patients with colorectal cancer (CRC), iron deficiency is highly prevalent, with prior reports citing a rate of 60% [3, 4]. Iron deficiency anemia in CRC patients occurs when iron stores are depleted due to factors such as malabsorption, tumor-induced anorexia, malnutrition and occult blood loss [3, 5]. Anemia can also occur as a result of chemotherapeutic agents [6]. Iron deficiency anemia, caused by chronic gastrointestinal bleeding, can lead to depletion of iron stores and absolute iron deficiency [7]. A separate entity is known as functional iron deficiency, where iron stores may be adequate within the body, but iron remains inaccessible for erythropoiesis due to an ongoing inflammatory state [7]. It is critical to diagnose and manage IDA among CRC patients since untreated IDA can adversely impact oncological outcomes. IDA is associated with more advanced clinical stage in colorectal cancer and worse disease free survival in some analyses [8]. CRC patients who have IDA also experience high levels of fatigue, which significantly impacts their perceived quality of life [9].

Traditionally, IDA has been treated through oral iron supplementation due to its low costs and convenience [10]. However, oral iron supplementation has been associated with an increase in gastrointestinal side effects [11], and nearly 95% of oral iron is excreted among CRC patients [12]. A recent study showed that administering oral iron supplements on alternate days and in single doses is an optimal dosing regimen [13]. In addition, intravenous iron has been commonly used to treat IDA among patients with CRC [3]. Intravenous iron allows for rapid normalization of total body iron even with one infusion [14], is less bioavailable to tumor cells and hence, not likely to increase the risk of tumor growth in CRC patients [15].

IDA can be difficult to diagnose at times, particularly in the presence of chronic inflammation, as may occur in the malignant setting [16, 17]. The diagnosis of IDA occurs when a patient is anemic with low hemoglobin (Hg) levels (< 12 g/dL in females; < 13 g/dL in men) in addition to iron deficiency, commonly shown through low ferritin levels [18, 19]. Among patients with clinical evidence of inflammation, serum ferritin levels below 100 μg/L should be considered [20]. In addition to using ferritin levels as a screening tool, other studies have recommended testing for iron saturation levels [19, 21, 22]. Nonetheless, IDA seems to be both under-diagnosed and under-treated [23].

While the impact of IDA in cancer patients has been well-documented, there is limited data regarding the proportion of CRC patients who are clinically screened and assessed for IDA. We aimed to study this knowledge gap by examining the proportion of CRC patients who get formally diagnosed for IDA and assess predictors of a formal diagnosed case of IDA, using data from electronic medical records (EMRs). We hypothesized that few patients were screened for IDA and received iron therapy.

## Methods

### Aim, research design and setting

This study aimed to assess the proportion of CRC patients who are tested for IDA and factors that predict a formal diagnosis of IDA and receiving iron therapy. The current study used a retrospective cross-sectional secondary analysis of EMRs of 499 colorectal cancer patients ages 18 and older. We abstracted data from the EMR system of a comprehensive cancer center in the Northeastern part of the United States. The authors’ Institutional Review Board approved the study.

### Patient and EMR selection

The research team worked with the Clinical Informatics division at the cancer center to select and compile EMR data of potentially eligible participants. The following inclusion criteria were considered: ages 18 and older, not deceased, diagnosed with CRC, seen at the cancer center within the past year, resident of the state, and English as their primary language. The lead author and a trained research assistant abstracted information regarding IDA diagnosis, hematology lab tests, iron supplementation, nutritional advice or support given to patients, stage of disease, smoking and drinking history, and sociodemographic characteristics. Abstracted data were double coded for the first twenty participants and randomly for the rest of the participants. Inconsistencies in data abstraction were corrected accordingly.

### Measures

The main outcome variable was a formal diagnosed case of IDA as entered by clinical staff in the EMRs. A formal diagnosis of IDA for each patient was ascertained via review of patient problem lists using International Classification of Diseases (ICD) 9/ICD 10 codes, medical history diagnoses, and manual review of documentation within recent clinical notes.

We classified participants as having absolute iron deficiency (yes/no) based on ferritin levels lower than 100 μg/L and iron saturation less than 20% [22]. We categorized participants as having functional iron deficiency based on ferritin levels between 100 to 500 μg/L and iron saturation less than 20% [19]. We looked at the latest laboratory results on ferritin, percentage iron saturation, iron, total binding iron capacity (TIBC), and hemoglobin (Hgb) levels that were available for all patients. In most cases, we could not draw conclusions about whether iron testing was intended to be part of routine care or initiated based on symptomatology because documentation was limited.

Potential covariates were selected based on previous research that demonstrated a link between these covariates and IDA and/or colorectal cancer [19, 24]. Covariates included sociodemographic factors, risky health behaviors, stage of CRC, and dietary variables. Sociodemographic covariates included gender (female, male); age groups (20–40, 41–60, 61–80, > 80); race categories (White, Black/American, Asian, Other); ethnicity (Hispanic/Latino, Non-Hispanic/Non-Latino), and marital status (single, married/have a partner, divorced/separated, widowed). Risky health behavior covariates included alcohol consumption status (non-drinker, current drinker, former drinker) and smoking status (non-smoker, current, former). We included stages 0 through II and stages III to IV as covariates in the regression analysis. Dietary covariates included whether patients received iron therapy (yes, no) and got nutritional counseling (yes, no).

### Data analysis

A large proportion of data that would be included on patients’ iron panel were missing, including that on ferritin (68.5%), percentage iron saturation (68.1%), TIBC (68.1%), iron (67.7%), and Hg (3.6%) (Table 1). About 25.9% of data were missing for stage of CRC cancer. Descriptive statistics were run for the study population. Most biological variables are not normally distributed [25], so the median and interquartile ranges (IQR) were reported for iron panel results and Hgb. For both outcomes on IDA diagnosis and receiving iron therapy, predictors included sex, race, marital status, alcohol consumption, smoking status, and getting nutritional counseling. Based on preliminary tests, we excluded age and ethnicity due to multicollinearity. We performed all statistical analyses by using the IBM Statistical Package for the Social Sciences version 28 (SPSS Inc., Chicago IL, US). Statistical tests were two-tailed with a level of significance of 0.05 (*p* < 0.05).

**Table 1:** Laboratory test values of participants (*N* = 499)

Lab tests (normal ranges)	n (%)	Missing (%)	Median	IQR
Ferritin, ng/mL (70–300)	157 (31.5)	342 (68.5)	65.0^a^	139.0
Iron Saturation, % (20–50)	159 (31.9)	340 (68.1)	13.0^a^	14.0
Iron, μg/dL (60–170)	161 (32.3)	338 (67.7)	39.0^a^	37.5
Total Iron Binding Capacity, μg/dL (240–450)	159 (31.9)	340 (68.1)	320.0	100.0
Hemoglobin, g/dL (> 12 for women; > 13 for men)	481 (96.4)	18 (3.6)	13.1 (men) 11.9^a^ (women)	2.8 2.5

*IQR* Interquartile range; ^a^Not within the normal ranges

## Results

### Patient characteristics

Table 2 shows the baseline characteristics of patients whose EMRs were abstracted. Most participants were aged between 61 and 80 (47.9%), White (70.7%), married or had a partner (58.9%). One hundred and thirty-two patients (26.5%) had stage III CRC while 102 had stage IV (20.4%). More than half of participants were current consumers of alcohol (51.1%). About 57% of participants were never-smokers while about 34% of them were former smokers. Only 98 (19.6%) of participants received nutritional support or advice.

**Table 2:** Characteristics of participants by IDA status (*N* = 499)

Participants Characteristics	Total		IDA (Yes)		IDA (No)			
	n	%	n	%	n	%	χ^2^	*p*
*Gender*							1.0	.314
Female	240	48.1	15	6.3	225	93.8		
Male	259	51.9	11	4.2	248	95.8		
*Age*							2.1	.548
20–40	16	3.2	0	0.0	16	100.0		
41–60	197	39.5	12	6.1	185	93.9		
61–80	239	47.9	13	5.4	226	94.6		
> 80	47	9.4	1	2.1	46	97.9		
*Race*							7.9	.047
White	353	70.7	15	4.2	338	95.8		
Black/African American	56	11.2	7	12.5	49	87.5		
Asian	47	9.4	1	2.1	46	97.9		
Other	2	0.4	0	0.0	2	100.0		
Unknown	41	8.2						
*Ethnicity*							1.6	.204
Hispanic/Latino	29	5.8	3	10.3	26	89.7		
Non-Hispanic/Non-Latino	467	93.6	23	4.9	444	95.1		
Unknown	3	0.6						
*Marital Status*							18.3	<.001
Single	94	18.8	9	9.6	85	90.4		
Married/Have a partner	294	58.9	8	2.7	286	97.3		
Divorced/Separated	53	10.6	8	15.1	45	84.9		
Widowed	47	9.4	1	2.1	46	97.9		
Unknown	11	2.2						
*Alcohol Consumption*							0.1	.962
Non-drinker	185	36.9	10	5.4	174	94.6		
Current	255	51.1	13	5.1	242	94.9		
Former	45	9.0	2	4.4	43	95.6		
Unknown	15	3.0						
*Smoking Status*							1.5	.466
Non-smoker	286	57.3	13	4.5	273	95.5		
Current	31	6.2	3	9.7	28	90.3		
Former	171	34.3	9	5.3	162	94.7		
Unknown	11	2.2						
*Disease Stage*							0.0	.839
0	1	0.2	1	0.2	0	0.0		
1	49	9.8	48	10.1	1	3.8		
2	86	17.2	79	16.7	7	26.9		
3	132	26.5	118	24.9	14	53.8		
4	102	20.4	101	21.4	1	3.8		
Unknown	129	25.9	126	26.6	3	11.5		
*Received Iron Therapy*							102.8	<.001
Yes	63	12.6	20	31.7	43	68.3		
No	436	87.4	6	1.4	430	98.6		
*Received Nutritional Counseling*							0.9	.337
Yes	98	19.6	7	7.1	91	92.9		
No	401	80.4	19	4.7	382	95.3		

### IDA diagnosis

Of the 499 patients, 26 (5.2%) had a formal diagnosis of IDA written on their EMRs (Table 2). Only 153 (30.7%) patients had complete iron panel data available, including ferritin, iron saturation, serum iron, and TIBC levels. Among these 153 patients, 113 (73.9%) had iron deficiency. Seventy-six (67.2%) had absolute iron deficiency as shown by ferritin levels below 100 ng/mL and iron saturation less than 20% and 37 (32.7%) had functional iron deficiency as shown by ferritin levels between 100 and 500 ng/mL and iron saturation less than 20%.

### Iron panel data

Median Hgb levels were 13.1 (11.6 to 14.4) g/dL for men and 11.9 (10.6 to 13.1) g/dL for women (Table 1). The median (IQR) ferritin level was 65.0 (26.0 to 165.0) ng/mL. The median iron saturation level was 13.0 (8.0 to 22.0) %. The median iron and TIBC levels were 39.0 (24.5 to 62.0) μg/dL and 320.0 (268.0 to 368.0) μg/dL, respectively as shown in Table 1.

### Iron therapy

Of the 499 patients, sixty-three (12.6%) received iron therapy (Table 2), out of which 20 had formally documented IDA. Out of the 63 CRC patients who received iron therapy, 49 received intravenous iron in the form of either 750 mg of ferric carboxymaltose or 125 mg of ferrlecit and 15 received oral iron tablet prescription in the form of 325 mg of ferrous sulphate.

Among patients with laboratory iron deficiency (*n* = 113), 55 (48.7%) had documentation of receiving iron therapy. Forty-eight of the 76 patients (63.2%) who had absolute iron deficiency received iron therapy. Seven of the 37 patients (18.9%) who had functional iron deficiency received iron therapy.

### Associations between patient characteristics and a formal diagnosis of IDA

No significant associations were found between patient characteristics and a formal diagnosis of IDA (*p* > .05).

### Associations between patient characteristics and receiving iron therapy

As shown in Table 3, Black/African American individuals were more likely to receive iron therapy than White participants [OR = 3.51, 95% confidence interval (CI) 1.49 to 8.27; *p* < .01]. Participants who were current (OR = 0.39, 95% CI 0.19 to 0.83) and former drinkers (OR = 0.09, 95% CI 0.72 to 3.22) were more likely to receive iron therapy as well (*p* < .05). Participants who received nutritional counseling were more likely to receive iron therapy than those who did not receive any nutritional counseling (OR = 2.43, 95% CI 1.15 to 5.14; *p* < .05).

**Table 3:** Predictors of a formally diagnosed case of IDA and receiving iron therapy among participants (*N* = 314)

	Adjusted model for IDA Diagnosis		Adjusted model for Receiving Iron Therapy	
Variable	OR	95% CI	OR	95% CI
*Gender*				
Female (ref)	–	–	–	–
Male	0.52	[0.17, 1.54]	1.34	[0.66, 2.72]
*Race*				
White (ref)	–	–	–	–
Black/African American	2.95	[0.95, 9.20]	3.51**	[1.49, 8.27]
Asian	0.63	[0.07, 5.31]	0.74	[0.23, 2.37]
*Marital Status*				
Single (ref)	–	–	–	–
Married/Have a partner	0.47	[1.39, 1.57]	0.79	[0.34, 1.83]
Divorced/Separated	1.59	[0.37, 6.76]	1.04	[0.29, 3.81]
Widowed	0.23	[0.02, 2.18]	1.12	[0.32, 3.86]
*Disease Stage*				
Stages 0 to II (ref)	–	–	–	–
Stage III to IV	1.10	[0.39, 3.09]	1.50	[0.74, 3.05]
*Alcohol Consumption*				
Non-drinker (ref)	–	–	–	–
Current	0.58	[0.18, 1.81]	0.39*	[0.19, 0.83]
Former	1.18	[0.20, 7.03]	0.09*	[0.01, 0.77]
*Smoking Status*				
Non-smoker (ref)	–	–	–	–
Current	2.00	[0.39, 10.25]	0.81	[0.19, 3.46]
Former	1.19	[0.37, 3.83]	1.52	[0.72, 3.22]
*Received Nutritional Counseling*				
No (ref)	–	–	–	–
Yes	1.71	[0.54, 5.46]	2.43*	[1.15, 5.14]

*OR* Odds ratio. *CI* Confidence interval

*p < .05. **p < .01. Category of “Other” for race was removed because of a very small number of cases. Age and ethnicity were not included in the regression model due to multicollinearity

Although it was not the purpose of this study and power was limited, secondary analyses were done to assess predictors of receiving nutritional support or advice. Male patients were more likely than their female counterparts to receive nutritional advice (OR = 0.46, 95% CI 0.25 to 0.86; *p* < .05). Current drinkers were more likely to receive nutritional advice than non-drinkers (OR = 2.36, 95% CI 1.16 to 4.78; *p* < .05). Those who were diagnosed with stages III to IV had higher odds of receiving nutritional advice than patients with stages 0 through II (OR = 2.47, 95% CI 1.27 to 4.78; *p* < .01). Patients who received iron therapy were also more likely to get nutritional advice than those who did not (OR = 2.33, 95% CI 1.11 to 4.91; *p* < .05).

## Discussion

Despite the high prevalence of IDA in CRC patients [3], clinical screening and assessment for IDA in CRC patients has been largely under-studied. This study examined the proportion of CRC patients who are formally diagnosed by an oncologist as documented in their EMRs and assessed predictors of a formal diagnosis of IDA and receiving iron therapy. The main findings indicated that only 26 (5.2%) CRC patients were formally diagnosed by an oncologist in their EMRs and only 153 (30.7%) of CRC patients had iron panel results available. These results suggest that IDA is likely to be under-diagnosed and under-documented in clinical notes despite the prevalence of IDA being up to 60% in CRC patients [4] and medical experts’ recommendations that all cancer patients be tested for iron deficiency [26]. Testing for and treatment of IDA is critical as it can improve overall quality of life and survival [2], prognosis [9], and reduce complications related to cancer treatment [26].

It is important to note that although 26 (5.2%) participants had a documented diagnosis of IDA, 63 (12.6%) received iron therapy. Thus, some patients were treated for IDA without a documented diagnosis (*n* = 43) while some were diagnosed with IDA without any documented treatment (*n* = 6). Twenty patients with formally documented IDA received iron treatment. Among those with available laboratory deficiency, patients with absolute iron deficiency (*n* = 48) were more likely to receive iron therapy than those with functional iron deficiency (*n* = 7). Functional iron deficiency, also known as anemia of chronic disease, is commonly found in cancer patients [5] and is characterized by insufficient availability of iron despite filled iron stores [6]. Although regular monitoring of iron status is recommended as part of standard of care for CRC patients [27], patients with functional iron deficiency might go untreated due to available iron stores showing up in laboratory results. In addition, it is plausible that some patients were iron deficient without any signs of anemia given that iron deficiency has been reported to be an independent risk factor for poor health outcomes among CRC patients [3]. Moreover, when considering supplements and OTC medications such as oral iron tablets, these may be under-reported in EMRs due to lack of inquiry from medical providers, perception of disclosure of medication as unimportant, fear of provider disapproval, among others [28].

We did not find any associations between patient characteristics and a formal diagnosis of IDA. In the present study, current and former alcohol drinkers were more likely to receive iron therapy for treating IDA. Chronic alcohol consumption suppresses hematopoiesis and dysregulates iron metabolism [29], thus potentially leading to IDA over time. In addition, former drinkers are likely to have poor health [30] and inadequate nutrition [31]. Hence, these factors can put former and current drinkers at increased need for IDA treatment. Not surprisingly, participants who received nutritional counseling were more likely to receive IDA treatment and vice-versa. Previous research has demonstrated the efficacy of nutritional counseling on symptom management in CRC patients [24].

A major advantage of this study is the use of EMRs which allowed access to different types of patient data including objective laboratory results. Two independent coders reviewed patient data at random to check for errors. Limitations include incomplete patient data in the EMRs and the lack of uniformity in how the data were documented in the EMRs. It is also possible that data missing in the EMRs were available elsewhere. Another limitation towards obtaining a formal diagnosis of IDA could be that some providers test for and document the condition more frequently than others. Additionally, the study was limited to one comprehensive cancer center and patients whose primary language was English. Some categories of variables included very small proportions for analysis. Therefore, we should be careful about making a conclusive claim for some variables. Lastly, this study design was cross sectional, so we cannot establish causality. We did not follow-up patients for long-term survival and perioperative outcomes and examine the differences in these outcomes among patients with or without IDA as it was beyond the scope of the current study.

### Implications for research and practice

Despite the high prevalence of IDA among CRC patients, only 30.7% of patients had available iron panel results and only 5.2% were formally diagnosed with IDA by an oncologist. This suggests that many CRC patients with IDA may go untreated. More research is needed on provider and system-level strategies that could improve recognition of this condition among CRC patients. In addition, it will be important for future studies to assess factors such as the design and organization of the EMRs which could improve patient data quality and documentation. From a practical perspective, oncologists and other providers in contact with CRC patients can integrate diagnosis of IDA as part of their routine cancer care so that all patients are frequently screened and given appropriate iron therapy.

## Conclusions

This study shows that, despite recommendations, less than 33% of CRC patients are clinically screened for IDA. Our findings illustrate the urgency of assessing complete iron panel data for all CRC patients. Integrating IDA screening in routine patient care for CRC patients will ensure that their condition is diagnosed and managed in a timely manner in addition to improving response to treatment, overall fatigue levels and survival. Future research should explore how to improve assessment and diagnosis of IDA in CRC patients at the provider level.

## Data Availability

The data is not publicly available because it contains potentially identifying information abstracted from cancer patients’ electronic medical records. The datasets used and/or analyzed during the current study are available from the corresponding author on reasonable request.

## Abbreviations

CRC: Colorectal Cancer
EMR: Electronic Medical Record
IDA: Iron Deficiency Anemia
OTC: Over the Counter
